# Bayesian neural networks for detecting epistasis in genetic association studies

**DOI:** 10.1186/s12859-014-0368-0

**Published:** 2014-11-21

**Authors:** Andrew L Beam, Alison Motsinger-Reif, Jon Doyle

**Affiliations:** Center for Biomedical Informatics, Harvard Medical School, Boston, MA USA; Bioinformatics Research Center, North Carolina State University, Raleigh, NC USA; Department of Statistics, North Carolina State University, Raleigh, NC USA; Department of Computer Science, North Carolina State University, Raleigh, NC USA

## Abstract

**Background:**

Discovering causal genetic variants from large genetic association studies poses many difficult challenges. Assessing which genetic markers are involved in determining trait status is a computationally demanding task, especially in the presence of gene-gene interactions.

**Results:**

A non-parametric Bayesian approach in the form of a Bayesian neural network is proposed for use in analyzing genetic association studies. Demonstrations on synthetic and real data reveal they are able to efficiently and accurately determine which variants are involved in determining case-control status. By using graphics processing units (GPUs) the time needed to build these models is decreased by several orders of magnitude. In comparison with commonly used approaches for detecting interactions, Bayesian neural networks perform very well across a broad spectrum of possible genetic relationships.

**Conclusions:**

The proposed framework is shown to be a powerful method for detecting causal SNPs while being computationally efficient enough to handle large datasets.

**Electronic supplementary material:**

The online version of this article (doi:10.1186/s12859-014-0368-0) contains supplementary material, which is available to authorized users.

## Background

The ability to rapidly collect and genotype large numbers of genetic variants has outpaced the ability to interpret such data, leaving the genetic etiology for many diseases incomplete. The presence of gene-gene interactions, or *epistasis*, is believed to be a critical piece of the “missing heritability” that is a hot topic in the field [1]. This has in turn spurred development on advanced computational approaches to account for these interactions, with varying degrees of success [2-4]. Recent work has shown that gene-gene interactions capable of influencing gene expression exist and are replicable [5], reinforcing the need for methodology that can account for these genetic interactions. The main computational challenge comes from the vast number of markers that are present in a typical association study. This problem is exacerbated when interactions between two or more markers must be considered. For example, given an experiment that genotypes 1,000 markers, examining all possible interactions between two of the markers involves consideration of nearly half a million combinations. This situation becomes exponentially worse as higher order interactions are considered. Modern genome-wide association studies (GWAS) routinely consider 1-2 million single nucleotide polymorphisms (SNPs), which would require examining half a trillion potential interactions. As whole genome sequencing (WGS) methods become commonplace, methods that cannot cope with large data sets will be of little utility. Data on this scale will require approaches that can find interactions without having to enumerate all possible combinations.

Several distinct types of methods have emerged that attempt to address this challenge. Perhaps one of the most popular approaches from the last decade has been *Multifactor Dimensionality Reduction* (MDR) [6,7], and extensions of the method. MDR is a combinatorial search that considers all possible interactions of a given order and selects the best model via cross validation. Because MDR is an exhaustive search, it suffers from the previously discussed scalability issue, though recent work using graphics processing units has attempted to lessen this deficit [8]. Though MDR is a general constructive-induction algorithm that can be paired with any stochastic search technique, it is most often paired with a permutation testing strategy to assess statistical significance for each marker, so the computational burden becomes prohibitive for large datasets. Permutation testing computes a p-value for a statistic of interest (such as an accuracy measure from MDR) by randomly permuting the class labels and calculating the statistic on the permuted dataset. This procedure is repeated many times to compute a “null” distribution for the statistic of interest. The percentage of instances in the permuted null distribution that are less than or equal to the actual statistic from the unpermuted data is taken as the desired one-sided p-value. Unfortunately, this can be extremely expensive for large datasets when many hypotheses are simultaneously tested, leading to a large multiple testing scenario. To get the required resolution for a Bonferroni corrected p-value of 0.05 when considering a set of 1,000 SNPs, one must perform 20,000 permutations. This makes permutation testing infeasible for even moderately sized datasets.

Another popular approach is Bayesian Epistasis Association Mapping (BEAM) [9]. BEAM partitions markers into groups representing individual (i.e. marginal) genetic effects, interactions, and a third group representing background markers that are uninvolved with the trait. BEAM employs a stochastic Markov Chain Monte Carlo (MCMC) search technique to probabilistically assign markers to each group and uses a novel “B-statistic” based on the MCMC simulation to assign statistical significance to each marker. This allows BEAM to assign statistical significance without the need to perform a costly permutation test. This method has been demonstrated successfully on data sets with half a million markers. However, the recommended amount of MCMC iterations needed is quadratic in the number of SNPs considered [9], possibly limiting its effectiveness for larger datasets.

Many popular machine-learning algorithms have also been adopted for use in analyzing association studies. Notable examples are decision trees (both bagged, i.e. random forests, [10,11] and boosted [12]) support vector machines (SVM) [10], Bayesian networks [13], and neural networks [2]. In particular, tree-based methods such as random forests and boosted decision trees have been found to perform well in a variety of association study analyses [11,12,14]. Machine learning approaches are appealing because they assume very little *a priori* about the relationship between genotype and phenotype, with most methods being flexible enough to model complex relationships accurately. However, this generality is something of a double-edged sword as many machine learning algorithms function as black boxes, providing investigators with little information on which variables may be most important. Typically it is the goal of an association study to determine which variables are most important, so a black box may be of little use. Some approaches have easy adaptations that allow them to provide such measures. Both types of tree based methods (bagged and boosted) can provide measures of relative variable importance [15-17], but these indicators lack measures of uncertainty, so they are unable to determine how likely a variable’s relative importance score is to occur by chance without resorting to permutation testing.

In this study, we propose the use of Bayesian neural networks (BNNs) for association studies to directly address some the issues with current epistasis modeling. While BNNs have been previously developed and applied for other tasks [18-21], they have yet to see significant usage in bioinformatics and computational biology. Like most complex Bayesian models, BNNs require stochastic sampling techniques that draw samples from the posterior distribution, because direct or deterministic calculation of the posterior distribution is often intractable. These posterior samples are then used to make inferences about the parameters of the model or used to make predictions for new data. Standard MCMC methods that employ a random walk such as the Metropolis-Hastings (RW-MH) algorithm [22,23] (which is the algorithm that forms the core of BEAM [9]) explores the posterior distribution very slowly when the number of predictors is large. If *d* is the number of parameters in a model, the number of iterations needed to obtain a nearly independent sample is O(*d*^*2*^) [24] for RW-MH. This makes the RW-MH algorithm unsuitable for neural network models in high-dimensions, so the Hamiltonian Monte Carlo (HMC) algorithm is instead used to generate samples from the posterior. HMC has more favorable scaling properties, as the number of iterations needed is only O(*d*^*5/4*^) [24]. HMC achieves this favorable scaling by using information about the gradient of the log-posterior distribution to guide the simulation to regions of high posterior density. Readers familiar with standard neural network models will notice an inherent similarity between Bayesian neural networks sampled using HMC and traditional feed-forward neural networks that are trained using the well-known back-propagation algorithm [25], as both take steps in the steepest direction using gradient based information. Though HMC will in general explore the posterior distribution in a more efficient manner than RW-MH, the evaluation of the gradient can very expensive for large data sets. Recent work has shown that this drawback can be lessened through the use of parallel computing techniques [26]. We demonstrate that a graphics processing unit (GPU) computational framework can enable the use of BNNs on large datasets.

The BNN framework outlined here has several features designed to address many of the challenges inherent in analyzing data from association studies. These advantages are outlined below.

- Quantification of variable influence with uncertainty measures. This allows variable influence to be assessed relative to a null or background model using a novel Bayesian testing framework. This avoids reliance on a permutation testing strategy.
- Automatic modeling of arbitrarily complex genetic relationships. Interactions are accounted for without having to examine all possible combinations. This is achieved from the underlying neural network model.
- An efficient sampling algorithm. HMC scales much better than other MCMC methods, such as the RW-MH algorithm, in high-dimensions.
- Computational expediency through the use of GPUs. The time needed to build the model is greatly reduced using the massive parallel processing offered by GPUs.

We offer evidence for these claims using several simulated scenarios and a demonstration on a real dataset. In addition, we compare the proposed approach to several popular methods so that relative performance can be assessed.

## Results and discussion

### Existing methods used for comparison

We selected several methods to serve as baselines for evaluation of the BNN’s performance. As previously mentioned BEAM and MDR are widely used methods and so were included in our evaluation. We used a custom compiled 64-bit version of BEAM using the source provided on the website [27] of the authors of [9]. The java-based MDR package was downloaded from the MDR source-forge repository (http://sourceforge.net/projects/mdr/) and called from within a Python script. To evaluate the effectiveness of tree-based methods, we used an approach nearly identical to that in [12], which was based on boosted decision trees. The boosted decision tree model provides a measure of *relative influence* for each variable that indicates how important a given variable is, relative to the others in the model. To fit the boosted tree model we used the *gbm* package in R. Finally, we also included the standard 2 degrees-of-freedom chi-square test of marginal effects.

As discussed, some approaches such as MDR and GBM require a permutation testing strategy to assess statistical significance. This makes assessing their performance on large datasets difficult, due to the amount time required to perform the permutation test. During our pilot investigations on a dataset containing 1,000 SNPs, each individual run of MDR was found to take roughly 1 minute to complete. The time needed to complete the required 20,000 permutations would be roughly 2 weeks. If we wish to evaluate a method’s effectiveness on hundreds or thousands of such datasets, this run time becomes prohibitive. As such, we divided our primary analysis into two sections. In the first section, we evaluated methods that do not rely on permutation testing on datasets containing 1,000 SNPs each. However, since we wish to compare the results of the BNN to that of MDR and GBM, we performed a second set of analyses on smaller datasets that only contained 50 SNPs each, for which permutation testing is feasible. This two-pronged strategy allowed us to evaluate a wide range of popular approaches in a reasonable amount of time, while serving to underscore the need for methods that do not rely on permutation testing.

### Parametric models of multi-locus relationships

In this section we performed an analysis of three biallelic models of genotypic relationships. These models have been used previously [9,12] and are meant to reflect theoretical and empirical evidence for genetic relationships involving multiple loci [28]. Tables 1, 2, and 3 contain the relative risk of disease for each genotype combination. Capital and lower case letters represent the major and minor alleles, respectively.

**Table 1:** **Additive risk model**

**Genotype**	**AA**	**Aa**	**aa**
**BB**	η	η(1 + θ)	η(1 + 2θ)
**Bb**	η(1 + θ)	η(1 + 2θ)	η(1 + 3θ)
**bb**	η(1 + 2θ)	η(1 + 3θ)	η(1 + 4θ)

**Table 2:** **Threshold risk model**

**Genotype**	**AA**	**Aa**	**aa**
**BB**	η	η	η
**Bb**	η	η(1 + θ)	η(1 + θ)
**bb**	η	η(1 + θ)	η(1 + θ)

**Table 3:** **Epistatic risk model**

**Genotype**	**AA**	**Aa**	**aa**
**BB**	η	η	η(1 + 4θ)
**Bb**	η	η(1 + 2θ)	η
**bb**	η(1 + 4θ)	η	η

The symbols η and θ in the tables represent the baseline risk and effect size, respectively. We simulated genotypes for the disease SNPs for a range of minor allele frequencies (MAFs) and simulated the disease status for 1,000 cases and 1,000 controls using the risks given in Tables 1, 2, and 3. We embedded the causal SNPs in a background of 998 non-causal SNPs, for a total of 1,000 SNPs to be considered. For each combination of effect size, *θ* ∈ {0.5, 1.0, 1.5, 2.0}, *MAF* ∈ {0.1, 0.2 , 0.3, 0.4, 0.5}, and model type (Additive, Threshold and Epistasis) we generated 100 datasets. This yielded a total of 6,000 datasets for evaluation. All datasets in this section were created using the R statistical programming language [29].

**Figure 1:**
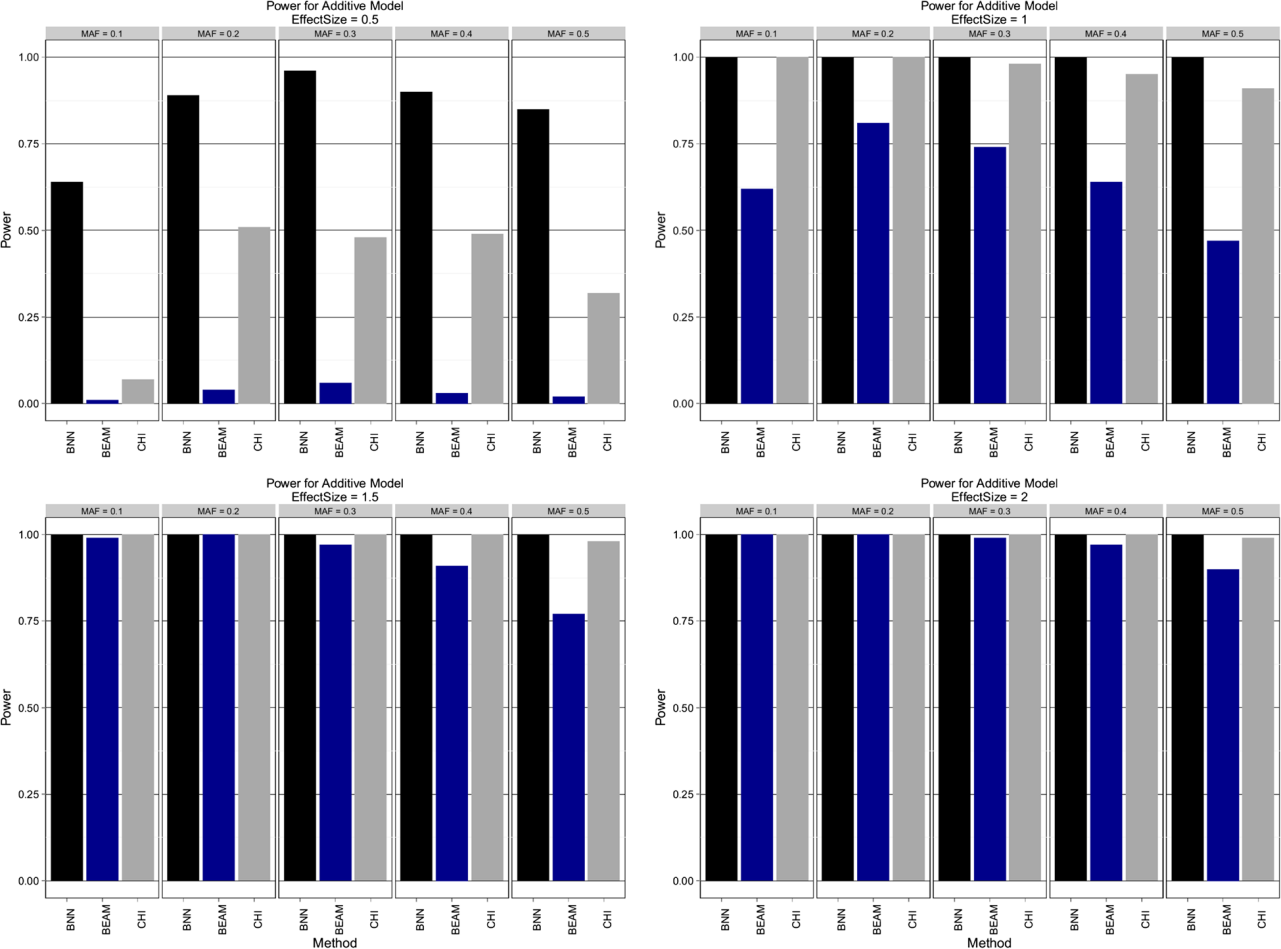
**Additive model.** Estimated power to detect both disease SNPs using Bayesian neural networks (BNN), BEAM, and *χ* ^2^ test (CHI) with 2 d.f. Effect sizes of {0.5, 1.0, 1.5, 2.0} are shown in order from left to right, top to bottom. Within each pane results are stratified by minor allele frequency (MAF).

**Figure 2:**
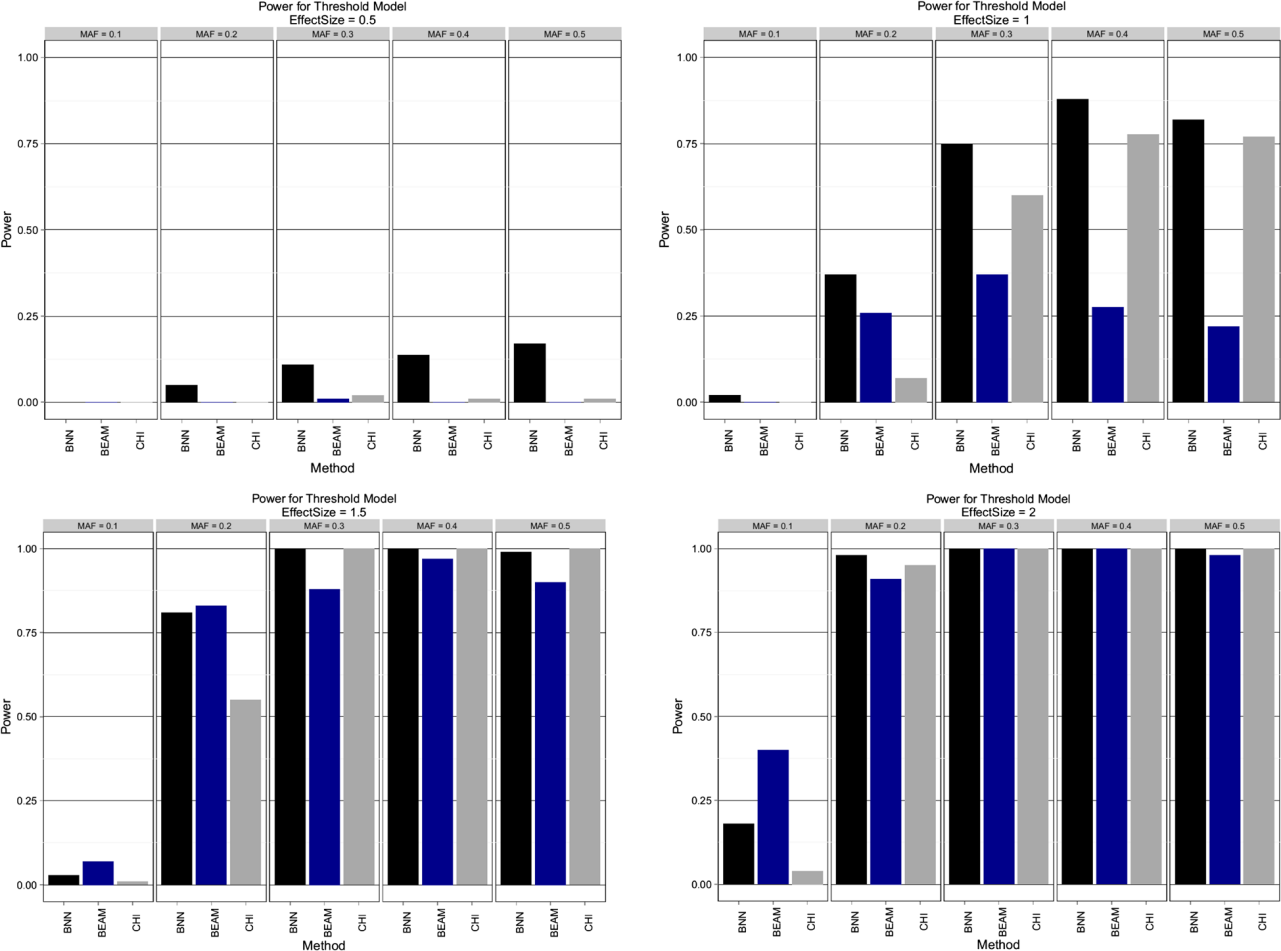
**Threshold model.** Estimated power to detect both disease SNPs using Bayesian neural networks (BNN), BEAM, and *χ* ^2^ test (CHI) with 2 d.f. Effect sizes of {0.5, 1.0, 1.5, 2.0} are shown in order from left to right, top to bottom. Within each pane results are stratified by minor allele frequency (MAF).

**Figure 3:**
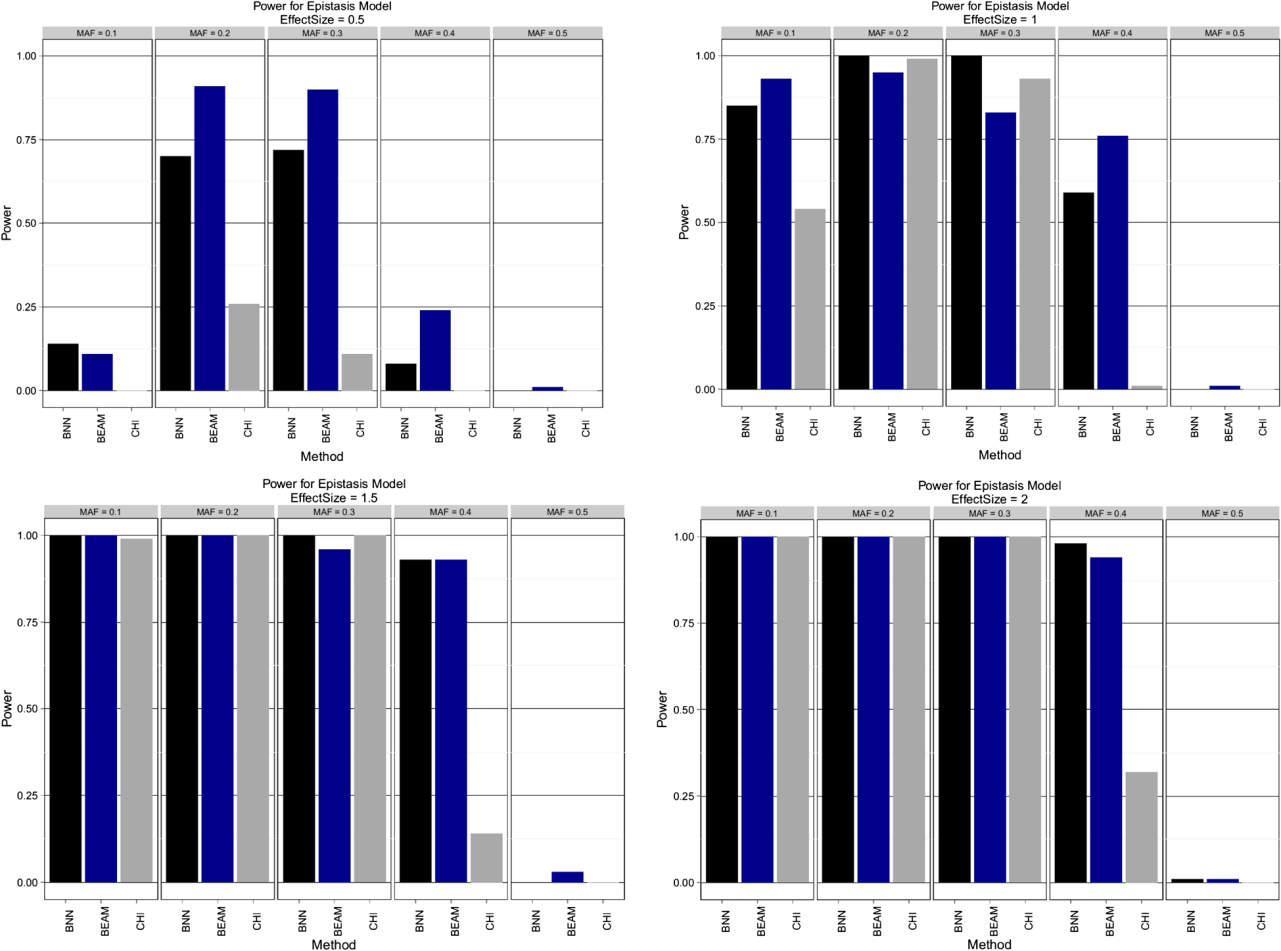
**Epistatic model.** Estimated power to detect both disease SNPs using Bayesian neural networks (BNN), BEAM, and *χ* ^2^ test (CHI) with 2 d.f. Effect sizes of {0.5, 1.0, 1.5, 2.0} are shown in order from left to right, top to bottom. Within each pane results are stratified by minor allele frequency (MAF).

We ran BNN, BEAM, and the *χ*^2^ test on each dataset and recorded whether or not both disease SNPs were declared as significant by each method. We took the fraction of datasets where both disease SNPs were correctly identified as an estimate of statistical power. For BEAM and the *χ*^2^ test, we used the canonical Bonferroni corrected significance threshold of p <0.05. We used the recommended parameter settings for BEAM [9] and performed 1e6 sampling iterations for each dataset. For the BNN approach, we used a network with 1 hidden layer and 5 logistic units and a softmax output layer with 2 units. The network parameters in the hidden layer are given ARD priors, while the network parameters in the output are given a common Gaussian prior. The hyper parameters for the Inverse-Gamma prior for the ARD parameters were *α*_0_ = 5, *β*_0_ = 2 while the hyper parameters for the Gaussian priors were *α*_0_ = 0.1, *β*_0_ = 0.1. The parameters for the HMC algorithm were ε = 5e-2, L = 15, α = 0.75, and T = 5e3. The cutoff value for the novel Bayesian ARD testing framework was 0.6. We discarded the first 25 samples as burn-in and kept 100 samples to be used for inference. Processing of each dataset by the BNN took approximately 3 minutes. The results are shown below in Figures 1, 2 and 3.

BNNs were found to be uniformly more powerful than both BEAM and the *χ*^2^ test for the additive model. BNNs show excellent power, even for small effect sizes and achieve 100% power for second smallest effect size across all tested MAFs. In contrast, BEAM showed relatively little power for the smallest effect size and never achieves 100% for all MAFs, even at the highest level of effect size. The threshold model tells a similar story. For all but 3 combinations of MAF and effect size, the BNN model is again uniformly more powerful than both BEAM and the *χ*^2^ test. The picture from the epistatic model is slightly more mixed. BEAM appeared to do a better job at the smallest effect size, while performing equally well as BNNs on the remaining three effect size levels. All three methods had almost no power to detect the causal SNPs for a MAF of 0.5. These results suggest that BNN is uniformly more powerful the *χ*^2^ test for these genetic models, and may be more powerful than BEAM in most instances.

### Simulated epistatic relationships without marginal effects

In this section, we evaluated the performance of all the methods examined in the previous section (BNN, BEAM, and the *χ*^2^) as well as GBM and MDR. Since MDR and GBM rely on permutation testing, we reduced the size of the dataset to accommodate this strategy. To generate test datasets, we used the GAMETES software package [30]. This package allows users to specify the proportion of variance for case/control status that is due to genetic variants (i.e. broad-sense heritability) as well as how many loci are involved in determining trait status. These relationships are generated such that there are minimal marginal effects, resulting in relationships that are nearly purely epistatic. Relationships without marginal effects are in some sense “harder” than those with marginal effects, because the causal SNPs contribute to trait status only through their interaction. Preliminary analysis on the reduced SNP datasets indicated that if the same models were used as in the previous section, most methods would have nearly 100% power for all simulated scenarios, which would provide little useful feedback for discerning which approaches were working best. This was the primary motivation for using the “harder”, purely epistatic relationships instead of the parametric models we used previously.

**Figure 4:**
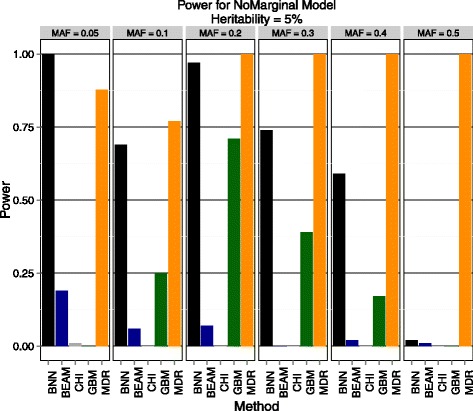
**Purely epistatic model with 5% heritability.** Estimated power to detect both disease SNPs of Bayesian neural networks (BNN), BEAM, *χ* ^2^ test (CHI) with 2 d.f., gradient boosted trees (GBM), and MDR. The results are stratified by minor allele frequency (MAF).

**Figure 5:**
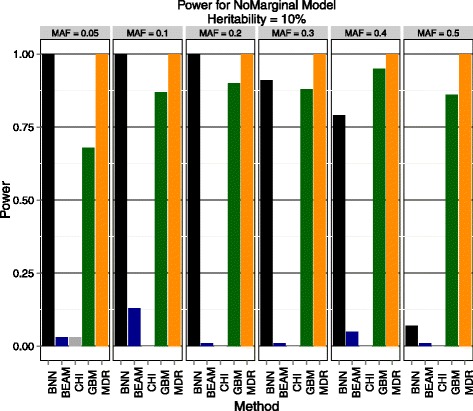
**Purely epistatic model with 10% heritability.** Estimated power to detect both disease SNPs of Bayesian neural networks (BNN), BEAM, *χ* ^2^ test (CHI) with 2 d.f., gradient boosted trees (GBM), and MDR. The results are stratified by minor allele frequency (MAF).

Using GAMETES, we analyzed two levels of heritability (5% and 10%) across a range of MAF (0.05, 0.1, 0.2, 0.3, 0.4, 0.5). Power was measured as in the previous section using 100 instances for each heritability/MAF combination for a total of 1200 data sets used in evaluation. The results are shown below in Figures 4 and 5.

BNN outperformed all methods from the previous section (BEAM and *χ*^2^ test) by a very wide margin. This suggests that BEAM may be less robust to detect causal SNPs in the absence of marginal effects than previously thought, as it never achieves 25% power in any of the scenarios tested. Again, we find these results encouraging as they indicate that BNNs are indeed powerful relative to existing approaches. Additionally, BNN outperformed the GBM method in all but 2 scenarios, indicating that BNN maybe be more adept at detecting purely epistatic signals across a broad array of MAFs and effect sizes. MDR performs well across every parameter combination tested, but as mentioned previously it is incapable of performing this analysis on a GWAS scale due to the exhaustive search technique and the need to perform permutation testing to assess statistical significance. To conclude this section, we note that BNN was the only method that did well across a variety of genetic models, number of SNPs, and MAFs while being capable of scaling to GWAS-sized data. This provides evidence that BNN framework is deserving of further investigation as an analysis technique for association studies.

### Sensitivity and specificity analysis of the ARD test

**Figure 6:**
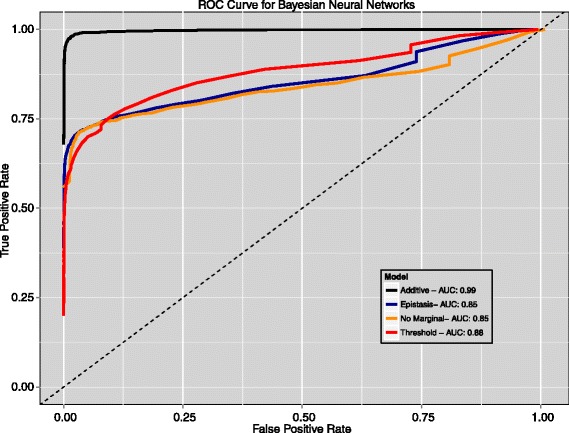
**Receiver-Operator Characteristic (ROC) curve for BNNs.** Each line represents the ROC curve for a different genetic model, averaged over effect size and MAF. The area under the curve (AUC) for each model is shown in the legend.

The cutoff value used for the ARD test has an obvious impact on the method’s performance. In the extreme case, a cutoff of 0 would result in nothing being significant while a cutoff value of 1 would result in everything being declared as such. The cutoff value controls the tradeoff between sensitivity (i.e. the true positive rate) and specificity (i.e. the true negative rate, which is equivalent to 1 – the false positive rate). Evaluation of the false positive rate for the cutoff value of 0.6 used in the previous experiments indicates that the BNN method properly controls the amount of false positives. We observed an average false positive rate (FPR) of roughly 0.005 and 0.06 for the parametric models and the purely epistatic models, respectively (see Additional file 1). To examine the trade off between the true positive rate (TPR) and FPR as the cutoff value is changed, we modulated the cutoff from 0 to 1 in increments of 0.01 and recorded the true positive and false positive rate for each data set in the two previous sections. In Figure 6, we averaged the TPR and FPR over effect size and MAF to produce a receiver-operator characteristic (ROC) curve for each of the 4 genetic models. The legend displays the area under the curve (AUC) for each model.

These results show that BNN-ARD test for variable importance is able to achieve a high true positive rate, while maintaining a low false positive rate, indicating the method is performing well and as expected.

#### Analysis of genome-wide tuberculosis data

To demonstrate the performance of Bayesian neural networks on a real dataset, we analyzed a GWAS designed to find genetic markers associated with tuberculosis (TB) disease progression. The dataset described in detail in [31], contains information on roughly 60,000 SNPS and 105 subjects, which we realize is a small sample size. For our study, each subject was classified as currently infected with any form of tuberculosis (i.e. extrapulmonary or pulmonary) or having a latent form of TB confirmed through a positive tuberculin skin test (purified protein derivative positive). Quality control was performed and SNPs with missing values were excluded, as were SNPs that were found to be out of Hardy-Weinberg equilibrium at the 0.05 level. After QC, there were 16,925 SNPs available for analysis and 104 subjects. Based on evidence of subpopulations in this data [31], subjects were assigned to one of three clusters created using the top two principal components and cluster membership was included as a covariate in the model. Sampling of the Bayesian neural network was conducted as outlined in the previous section, with ARD hyper-parameters of *α*_0_ = 3, *β*_0_ = 1. We performed 100 burn-in iterations followed by 1,000 sampling iterations, which took approximately 20 hours. The top five SNPs based on posterior ARD probabilities are shown below in Table 4. The time needed to perform the simulation is dependent upon both the dataset size and the desired number of simulations. Using this dataset as an example, the sampler completed one full HMC update approximately every minute, thus on a datasets of 500,000 and 1 million SNPs we would expect a sample every 30 minutes and every hour, respectively. We provide these numbers as rough estimates of the needed computational time per sample of data sets with larger numbers of SNPs.

**Table 4:** **Top 5 SNPs based on posterior ARD probabilities (a larger probability indicates a SNP is more likely to be involved)**

**SNP**	**CHR**	***Pr*** **(** ***μ***_***j***_ **>** ***μ***_***null***_ **)**
rs966414	2	0.524
rs1378124	8	0.515
rs9327930	5	0.509
rs4721214	7	0.502
rs10512384	9	0.498

The SNP reported as the 2^nd^ most significant in [31] (rs10490266) appeared in our analysis as the 31^st^ most significant SNP. Only one of the SNPs in Table 1 is currently known to be located within a gene (rs1378124 - MATN2) according to dbSNP. Every SNP reported in Table 4 is located on the same chromosome and within 10-50 MB of loci previously reported as having a statistically significant association with pulmonary tuberculosis susceptibility [32]. The loci reported in [32] were unfortunately either not part of the original SNP library or removed during the QC process in this study. Due to the small sample size of this dataset, it is hard to say conclusively which of the SNPs reported here and in [31] are most likely to replicate in a larger study. However, we present this analysis to demonstrate that the BNN framework is capable of analyzing data sets containing a high number of SNPs in a relatively short time.

**Figure 7:**
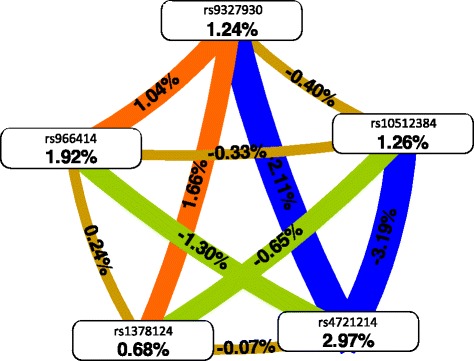
**Entropy network for the Top 5 SNPs selected by the BNN.**

Here we explore the types of interactions between the top 5 SNPs from the real data analysis using an entropy web. The purpose of the interaction web is to visually display the nature of the interactions (redundant, additive, or synergistic) amongst the 5 SNPs. The colors used comprise a spectrum of colors representing a continuum from Synergy to Redundancy. The colors range from red representing a high degree of synergy (positive information gain), orange a lesser degree, and gold representing the midway point between synergy and redundancy. On the redundancy end of the spectrum, the highest degree is represented by the blue color (negative information gain) with a lesser degree represented by green. The numbers indicate the entropy explained by each of the variables or variable combinations, with the weight of connections proportional to the strength of the signal. Positive numbers indicate synergy between variables, while negative number indicate redundancy. This information is displayed in Figure 7. The figure indicates that several of the SNPs are indeed weakly interacting with one another (rs9327930 with rs1378124 and rs9327930 with rs966414), giving confidence that the method is capable of detecting relevant SNPs in the presence of interactions.

## Conclusions

In this study we have proposed the use of Bayesian neural networks for association studies. This approach was shown to be powerful across a broad spectrum of different genetic architectures, effect sizes, and MAFs. Of the approaches that do not rely on permutation testing, BNN was uniformly more powerful than the standard *χ*^2^ test and almost uniformly more powerful than the popular BEAM method in the scenarios considered. BNN again showed a near uniformly better performance than the GBM method. MDR was very competitive with BNN in our evaluations, however MDR is incapable of scaling to larger datasets due to both its exhaustive search technique and reliance on permutation testing. In conclusion, we have demonstrated that BNNs are a powerful technique for association studies while having the capability of scaling to large GWAS sized datasets.

### Availability of code

The code implementing the GPU-based Bayesian neural network framework outlined in this paper is available at https://github.com/beamandrew/BNN.

## Methods

### Bayesian neural networks

Neural networks are a set of popular methods in machine learning that have enjoyed a flurry of renewed activity spurred on by advances in training so-called “deep” networks [33]. In the most basic sense, neural nets represent a class of non-parametric methods for regression and classification. They are non-parametric in the sense that they are capable of modeling any smooth function on a compact domain to an arbitrary degree of precision without the need to specify the exact relationship between input and output. This is often succinctly stated as “neural nets are *universal function approximators*” [34]. This property makes them appealing for many tasks, including modeling the relationship between genotype and phenotype, because a sufficiently complex network will be capable of automatically representing the underlying function.

Some drawbacks of classical neural nets are natural consequences of their strengths.

Due to their flexibility, neural nets are highly prone to “over-fitting” to data used to train them. Over-fitting occurs when the network starts to represent the training data exactly, including noise that may be present, which reduces its ability to generalize to new data. Many methods exist to address this issue, but popular methods such as weight decay are well known to be approximations to a fully Bayesian procedure [20,35]. Another issue with standard neural nets is they are often regarded as “black boxes” in that they do not provide much information beyond a predicted value for each input. Little intuition or knowledge can be gleaned as to which inputs are most important in determining the response, so nothing coherent can be said as to what drives the network’s predictions. Discussions of the advantages and disadvantages of NN for gene mapping have been reviewed in [36]. First we describe the base neural network model, and then describe how this can be incorporated into a Bayesian framework.

The network is defined in terms of a directed, acyclic graph (DAG) where inputs are feed into a layer of *hidden units*. The output of the hidden units are then fed in turn to the output layer which transforms a linear combination of the hidden unit outputs into a probability of class membership. Specifically consider a network with *p* input variables, *h* hidden units, and 2 output units to be used to predict whether an observation belongs to class 1 or class 2. Let x_*i*_ = < *x*_*i*1_, …, *x*_*ip*_ > be the input vector of *p* variables and y_*i*_ = < *y*_*i*1_, *y*_*i*2_ > be the response vector, where y_i1_ = 1 if observation *i* belongs to class 1 and 0 if not, with y_i2_ is defined in the same way for class 2. Hidden unit *k* first takes a linear combination of each input vector followed by a nonlinear transformation, using the following form:

1 $$ {h}_k\left({x}_i\right)=\phi \left({b}_k+{\displaystyle \sum_{j=1}^p{w}_{kj}*{x}_{ij}}\right) $$

where ϕ (.) is a nonlinear function. For the purposes of this study, consider the logistic transformation, given as:

$$ \phi (z)=\frac{1}{1+{e}^{-z\ }} $$

Several other activation functions such as the hyperbolic tangent, linear rectifier, and the radial basis/Gaussian functions are often used in practice. An output unit takes a linear combination of each *h*_*k*_ followed by another nonlinear transformation. Let *f*_1_(*x*_*i*_) be the output unit that is associated with class 1:

2 $$ {f}_1\left({x}_i\right)=\psi \left({B}_1+{\displaystyle {\sum}_{k=1}^h{W}_{k1}*{h}_k\left({x}_i\right)}\right) $$

Note we have used upper case letters to denote parameters in the output layer and lowercase letters to indicate parameters belonging to the hidden layer. The *ψ*(.) function is the *softmax* transformation of element z_1_ from the vector ***z***:

$$ \psi \left({z}_1\right)=\frac{ \exp \left({z}_1\right)}{{\displaystyle {\sum}_i} \exp \left({z}_i\right)} $$

*f*_1_(***x***_***i***_) represents the estimated conditional probability that y_i_ belongs to class 1, given the input vector x_*i*_. A similar definition is made for output unit 2, *f*_2_(*x*_*i*_). Note that for the case of only 2 classes, *f*_2_(*x*_*i*_) = 1 − *f*_1_(*x*_*i*_) because the softmax transformation forces the outputs to sum to 1.

Having described the formulation for standard neural networks we next describe how this can be extended using the Bayesian formulation. Bayesian methods define a probability distribution over possible parameter values, and thus over possible neural networks. To simplify notation, let *θ* = {*B*, *W*, *b*, *w*} represent all of the network weights and biases shown in equations (1), (2). The posterior distribution for θ given the data x_*i*_ and y_*i*_, is given according to Bayes’ rule:

3 $$ p\left(\theta \Big|{x}_i,{y}_i\right)=\frac{L\left(\theta \Big|{x}_i,{y}_i\right)\cdot \pi \left(\theta \right)}{m(x)} $$

where *m*(*x*) = ∫*L*(*θ*|*x*_*i*_, *y*_*i*_) ⋅ *π*(*θ*)*dθ* is the marginal density of the data. *L*(*θ*|*x*_*i*_, *y*_*i*_) is the *likelihood* of θ given the data and *π*(*θ*) is the prior distribution for the network parameters. However, in practice we only need to be able to evaluate the numerator of (3) up to a constant because we will be relying on MCMC sampling techniques that draw from the correct posterior without having to evaluate *m*(*x*), which may be intractable in high dimensions. In practice, it is often better to work with the log-likelihood *l*(*θ*|*x*_*i*_, *y*_*i*_) = log(*L*(*θ*|*x*_*i*_, *y*_*i*_)), because the raw likelihood can suffer from numerical overflow or underflow. In this study we assume the log-likelihood for a neural network with 2 output units is binomial:

4 $$ l\left(\theta \Big|{x}_i,{y}_i\right)={y}_{i1}\cdot \log \left({f}_1\left({x}_i\right)\right)+{y}_{i2}\cdot \log \left(1-{f}_1\left({x}_i\right)\right) $$

Next every parameter in the model must be given a prior distribution. The prior distribution codifies beliefs about the values each parameter is likely to take before seeing the data. This type of formulation is extremely useful in high-dimensional settings such as genomics, because it enables statements such as “most of the variables are likely to be unrelated to this response” to be quantified and incorporated into the prior. In this study, we adopt a specific prior structure known as the *Automatic Relevance Determination (ARD)* prior. This prior was originally introduced in some of the foundational work on Bayesian neural nets [20,37] and later used in other types of models [38].

The ARD prior groups network weights in the hidden layer together in a meaningful and interpretable way. All of the weights in the hidden layer that are associated with the same input variable are considered part of the same group. Each weight in a group is given a normal prior distribution with mean zero and a common variance parameter. This shared group-level variance parameter controls how large the weights in a group are allowed to become and performs *shrinkage* by pooling information from several hidden units, which helps to prevent overfitting [37,39]. Each of the group-level variance parameters is itself given a prior distribution, typically an Inverse-Gamma distribution with some shape parameter α_0_ and some scale parameter β_0_. These parameters often referred to as *hyper parameters*, can themselves be subject to another layer of prior distributions, but for the purposes of this study, we will leave them fixed as user specified values. Specifically, for a network with *h* hidden units, the weights in the hidden layer for input variable j will have the following prior specification:

$$ {w}_{j1},\dots, {w}_{jp}\sim N\left(0,{\sigma}_j^2\right) $$

$$ {\sigma}_j^2 \sim IG\left({\alpha}_0,{\beta}_0\right) $$

This structure allows the network to automatically determine which of the inputs are most relevant. If variable *j* is not very useful in determining whether an observation is a case or a control, then the posterior distribution for σ_j_^2^ will be concentrated around small values. Likewise, if variable j is useful in determining the response status, most of the posterior mass for σ_j_^2^ will be centered on larger values.

## Additional file

Additional file 1:
**Supplemental information [** 20,21,24,26,40 **-** 46 **].**
