# Supplementary material for: Bayesian neural networks for detecting epistasis in genetic association studies
Source: BMC Bioinformatics. 2014 Nov 21;15(1):368. doi: 10.1186/s12859-014-0368-0 (PMC4256933; doi:10.1186/s12859-014-0368-0)
Supplement: Additional file 1: — Supplemental information [ 20,21,24,26,40 - 46 ]. [file 12859_2014_368_MOESM1_ESM.docx]

# Supplemental Information

**Sensitivity and Specificity - Estimated False Positive Rate**

To examine the effect of the cutoff of 0.6 used during the simulated scenarios, we computed the false positive rate (FPR) for each dataset used in the previous sections. We averaged over the MAF values to produce FPRs for each combination of genetic model and effect size. These values are shown in Figure 8.

Figure 8 shows that for the additive, threshold, and epistatic models BNNs achieved a very low FPR of roughly 0.005 for all instances. For the more difficult models without marginal effects, the procedure still achieves a very good FPR of 0.06. This suggests that BNNs are both sensitive and specific in terms of having good power to detect true causal SNPs while avoiding large numbers of false positives.

**Hamiltonian Monte Carlo (HMC) for Neural Networks**

Here we briefly give an overview of Hamiltonian Monte Carlo (HMC) for neural networks, but please see [1-3] for a thorough treatment. HMC is one of many Markov Chain Monte Carlo (MCMC) methods used to draw samples from probability distributions that may not have analytic closed forms. HMC is well suited for high-dimensional models, such as neural nets, because it uses information about the gradient of the log-posterior to guide the sampler to regions of high posterior probability. For neural networks, we adopt the two-phase sampling scheme of Neal used in [1]. In the first phase, we update the values of the variance parameters using a Gibbs update, conditional on the current values of the network’s weights. In the next phase, we leave the variance parameters fixed and update the network weights using HMC. We repeat this procedure of Gibbs-coupled HMC updates until we have acquired the desired number of posterior samples.

The higher-level variance parameters, including those in the ARD prior, have simple closed forms because the Inverse-Gamma distribution is a conditionally conjugate prior distribution for the variance parameter of a Normal distribution. To obtain a new value for a variance parameter, conditional on the values of the weights a parameter controls, one makes a draw from the following Inverse-Gamma distribution:

$\sigma_{new}^{2}\sim IG\left( \alpha_{0}+\frac{n_{w}}{2},\beta_{0}+\sum_{i=1}^{n_{w}} \frac{w_{i}^{2}}{2} \right)$ (*)

where each w_i_ is a weight controlled by this variance parameter, n_w_ is the number of weights in the group, and α_0_, β_0_ are the shape and scale parameters respectively of the prior distribution.

HMC then proceeds by performing *L* “leap-frog” updates for the weights, given the values of the variance parameters. The algorithm introduces a momentum variable for every parameter in the network. The momentum variables and networks parameters will be updated by simulating Hamiltonian dynamics on the surface of the log-posterior density. HMC was originally created in statistical physics community, so it is often presented in terms of “energy potentials” which is equivalent to an exponentiation of the negative log-posterior, but we will describe the algorithm directly in terms of the log-posterior, which is more natural for our purposes. The full algorithm for sampling the posterior of all parameters, both network weights, variance parameters, is shown in detail below:

**HMC Algorithm for Neural Networks**

**Input:** **X**: matrix of predictors **Y**: matrix of class membership

**P**: log-posterior density to be sampled

{**ε**, **L**, **N_s_**, **α**}: HMC Parameters

**Output: N_s_** Posterior Samples of Model Parameters

BEGIN ALGORITHM:

θ_0_ = InitializeNetworkParameters()

σ_0_^2^ = InitializeVarianceParameters()

m_prev_ ~ N(0,1)

FOR i in 1 to N_s_ DO:

σ_i_^2^ = GibbsUpdate(θ_i-1_)

γ_0_ = θ_i-1_

m_0_ = InitializeMomentum(α, m_prev_)

FOR t in 1 to L DO:

END

m_prev_ = m_t_

IF Accept(γ_t_): θ_i_ = γ_t_

ELSE: θ_i_ = θ_i-1_

END

A few details in the HMC algorithm as shown need further explanation. First the momentum variables (*m*) are refreshed after every sequence of *L* leap-frog updates, shown in the algorithm as InializeMomentum(α). In the simplest formulation, each momentum component is an independent draw from a Normal distribution, with mean 0 and standard deviation of 1. However, this can lead to wasted computation because the sampler may start out in bad direction by chance, requiring many leap-frog updates until the sampler is heading in a useful direction, which could result in random-walk like behavior. To combat this, we use the persistent momentum refreshes [1, 4] which initializes the momentum using a weighted combination of the final momentum value of the previous leap-frog update and a draw of standard normal random variable. Using the notation of the algorithm, this is shown below

$\zeta\sim N\left( 0,1 \right)$
 $m_{0}=\alpha*m_{prev}+ \sqrt{1-\alpha^{2}}*\zeta$ (*)

However, if the proposal is rejected the momentum is negated. This must be done to ensure that the canonical distribution is left intact [4]. This formulation reduces the number of leap-frog updates (L) needed to reach a distant points by suppressing random-walk behavior and it leaves the correct stationary distribution of the Markov-chain intact [4].

The $Accept(\gamma_{t})$ function returns true if the new proposal, $\gamma_{t}$ is accepted according to a modified Metropolis-Hastings acceptance probability. $Accept(\gamma_{t})$returns true with the following probability:

$$\bar{\alpha}=min\left( 1,\frac{P\left( \gamma_{t} | X,Y;\sigma_{i}^{2} \right)-\frac{1}{2}m_{t}^{T}m_{t}}{P\left( \gamma_{t-1} | X,Y;\sigma_{i}^{2} \right)-\frac{1}{2}m_{t-1}^{T}m_{t-1}} \right)$$

In practice the posterior distribution is often very `bumpy' with many posterior modes [1]. This property may be exacerbated in high dimensions, so becoming stuck in one mode for extended periods of time is a great concern. To alleviate this, we modify the acceptance probability procedure in the following way. We instead sample from a *flattened* version of the posterior whose acceptance probability is given as $\alpha^{*}=\bar{\alpha}\cdot T$ for T > 1, which is equivalent to sampling from $P\left( \gamma| X,Y;\sigma_{i}^{2} \right)^{\frac{1}{T}}$ . While it is true that we are no longer sampling from the exact posterior $P(\gamma|X,Y;\sigma_{i}^{2})$, under mild regularity conditions the posterior modes of the correct distribution remain intact [5]. Since none of the parameters have biological interpretations modifying the posterior in this way of little concern, if the full procedure is capable of maintaining high sensitivity and specificity. We find the trade-off between ease of sampling across a wide-range possible scenarios and exactness of the posterior to be acceptable.

**HMC using Graphics Processing Units (GPUs)**

Previous work has shown how the gradient and log-posterior evaluations needed by HMC can be sped-up by as much as 150x for large problems using Graphics Processing Units (GPUs) [RW.ERROR - Unable to find reference:122]. We adopt that framework here and express the gradient calculations as matrix-vector operations or element-wise operations. Similarly, evaluation of the log-posterior can be expressed in terms of linear operators and element-wise operations. Using GPUs for these operations is well known in the neural network literature [6-8] as the gradient of the log-posterior corresponds roughly to the well-known “back-prop” algorithm and evaluation of the log-posterior corresponds to the “feed-forward” operation in standard neural networks. However, to our knowledge this study represents the first GPU-enabled implementation of Bayesian neural networks. Without GPU computing, it is likely that the computational burden imposed by genomic data would be too great for the Bayesian neural network framework to be feasible.

All of methods discussed in this study are implemented in the Python programming language. All GPU operations were conducted using the Nvidia CUDA-GPU [9] programming environment and accessed from Python using the PyCuda library [10]. Source code containing the Bayesian neural network package is available at https://github.com/beamandrew/BNN

**Bayesian Test of Significance for ARD Parameters**

Given the ARD prior a natural question to ask is how large do values of σ_j_^2^ need to be for input *j* to be considered relevant compared to a variable that is *completely* unrelated. This question can be framed in terms of a Bayesian hypothesis test. In this framework we will assume that under the “null hypothesis” a variable is completely irrelevant in determining the status of the response. If this were a simple linear model, this would be equivalent to saying the regression coefficient for this variable has a posterior mean of zero. In the neural network model the ARD parameters that determine how relevant each input is are strictly positive, so we need a baseline or null model for the ARD parameters in order to determine if we can “reject” this null hypothesis of irrelevance. In order to construct and test this hypothesis, we make a simplifying assumption that weights for unrelated variables have a normal distribution with mean 0 and variance $\sigma_{null}^{2}$ i.e. $w_{kj}\sim N(0,\sigma_{null}^{2})$. Due to the complex statistical model, the true posterior distributions for the weights under the null may not be exactly normal, but this approximation will be useful in simplifying the calculations. Additionally since, the prior for each weight is normal, this approximation will most likely not be too far from true posterior form.

Since $\sigma_{null}^{2}$ represents the “null” ARD parameter associated with a variable of no effect, we wish to test whether a variable of interest is “significantly” greater than this null value. We use the phrases “null” and “significance” here because of their familiar statistical connotations, but they should not be confused with the p-value based frequentist hypothesis testing procedure, as we are operating within a fully Bayesian framework. Our goal becomes testing whether the mean, $\mu_{j}$ of the posterior distribution for the ARD parameter, $\sigma_{j}^{2}$, is greater than the mean of the null, $\mu_{null}$, for the null ARD parameter $\sigma_{null}^{2}$. Specifically we wish to test the following null hypothesis:

$$H_{0}: \mu_{null}= \mu_{j}$$

against the one-sided alternative:

$$H_{a}: \mu_{null}<\mu_{j}$$

To test this, we need to know the closed form of $\mu_{null}$. Making use of the iterative two-stage sampling scheme, we will derive this form by induction. We will also make use of several well-known facts of random variables. Firstly, if a random variable X has an inverse-gamma distribution, i.e. $X\sim IG\left( \alpha,\beta\right)$, then the mean or *expected value* of X, E[X], is given by $\frac{\beta}{\alpha-1}$. Next, if variables $X_{1}, \ldots, X_{n}$are each independently and identically distributed as $N(0,\sigma^{2})$, then $\sum_{i=1}^{n} \left( \frac{X_{i}}{\sigma} \right)^{2}=<\frac{X_{1}}{\sigma}, \ldots,\frac{X_{n}}{\sigma}>^{T}<\frac{X_{1}}{\sigma}, \ldots,\frac{X_{n}}{\sigma}>\sim\chi_{n}^{2}$ ,i.e. a chi-squared random variable with *n* degrees of freedom. This sum has an expected value of *n*, from the definition of a chi-squared random variable. This implies the conditional expected value $E\left[ <X_{1}, \ldots, X_{n}>^{T}<X_{1}, \ldots, X_{n}>|\sigma\right]=\sigma^{2}n$. Using these basic facts we will show that under the null, the two-stage sampling scheme leaves expected value of the ARD parameter invariant, i.e. $\mu_{null}=\mu_{prior}$.

For a network with *h* hidden units, let $w_{j}= <w_{1j},\ldots, w_{hj}>$be a vector containing all of the weights associated with input *j*, where each component of $w_{j}$ is initially distributed according to the prior, $N\left( 0,\sigma_{j}^{2} \right)$ and $\sigma_{j}^{2}\sim IG\left( \alpha_{0},\beta_{0} \right)$. We begin the simulation at iteration i=1 and perform a Gibbs update of $\sigma_{j}^{2}$. The Gibbs update for the shape parameter, $\alpha_{1}=\alpha_{0}+\frac{n_{w}}{2}$, is iteration independent and will remain fixed for the entirety of the simulation. However, the Gibbs update for the scale parameter, $\beta_{1}= \beta_{0}+\frac{w_{j}^{T}w_{j}}{2}$, depends upon the current values of the weights, and thus will take on a random value at each iteration. However, we can compute the expected value for $\beta_{1}$ as:

$${E[\beta}_{1}]= E\left[ \beta_{0}+\frac{w_{j}^{T}w_{j}}{2} \right]$$

$${E[\beta}_{1}]=\beta_{0}+\frac{1}{2} E\left[ w_{j}^{T}w_{j} \right]$$

$$E\left[ \beta_{1} \right]=\beta_{0}+\frac{1}{2}(h*{E[\sigma}_{0}^{2}])$$

$$E\left[ \beta_{1} \right]=\beta_{0}+\frac{h}{2}*\frac{\beta_{0}}{\alpha_{0}-1}$$

$$E\left[ \beta_{1} \right]= \beta_{0}+\frac{h}{2}*\mu_{0}$$

Thus, the expected value of $\beta_{1}$after the first Gibbs update is $\beta_{0}+\frac{h}{2}*\mu_{0}$. Note that this expectation is independent of simulation iteration, so this result will hold for all $\beta_{1},\beta_{2},\ldots,\beta_{t}$. Next, we use this fact to compute the expected value of the ARD parameter, $E\left[ \sigma_{1}^{2} \right]$:

$$E\left[ \sigma_{1}^{2} \right]=E\left[ \frac{\beta_{1}}{\alpha_{0}+\frac{h}{2}-1} \right]$$

$$=\frac{E\left[ \beta_{1} \right]}{\alpha_{0}+\frac{h}{2}-1}$$

$$=\frac{\beta_{0}+\frac{h}{2}\mu_{0}}{\alpha_{0}+\frac{h}{2}-1}$$

$$=\frac{\beta_{0}}{\alpha_{0}-1+\frac{h}{2}}+\frac{\frac{h}{2}\mu_{0}}{\alpha_{0}-1+\frac{h}{2}}$$

$$=\frac{\frac{1}{\alpha_{0}-1}}{\frac{1}{\alpha_{0}-1}}\left( \frac{\beta_{0}}{\alpha_{0}-1+\frac{h}{2}} \right)+\frac{\frac{h}{2}\mu_{0}}{\alpha_{0}-1+\frac{h}{2}}$$

$$= \frac{\mu_{0}}{1+\frac{h}{2}*\frac{1}{\alpha_{0}-1}}+\frac{\frac{h}{2}\mu_{0}}{\alpha_{0}-1+\frac{h}{2}}$$

$$=\mu_{0}\left( \frac{1}{1+\frac{h}{2}*\frac{1}{\alpha_{0}-1}}+\frac{\frac{h}{2}}{\alpha_{0}-1+\frac{h}{2}} \right)$$

$$=\mu_{0}\left( \frac{\alpha_{0}-1}{\alpha_{0}-1+\frac{h}{2}}+\frac{\frac{h}{2}}{\alpha_{0}-1+\frac{h}{2}} \right)$$

$$=\mu_{0}\left( \frac{\alpha_{0}-1+\frac{h}{2}}{\alpha_{0}-1+\frac{h}{2}} \right)$$

$$=\mu_{0}$$

Thus, the Gibbs update of the ARD parameter does not change the expected value under the null, since we defined $E\left[ \sigma_{0}^{2} \right]=\mu_{0}$. This establishes the base case, and now we show the induction step. Given $E\left[ \beta_{t+1} \right]=E\left[ \beta_{t} \right]=\beta_{0}+\frac{h}{2}\cdot\mu_{0}$and $E\left[ \sigma_{t} \right]= \mu_{0}$then:

$$E\left[ \sigma_{t+1}^{2} \right]=E\left[ \frac{\beta_{t+1}}{\alpha_{0}+\frac{h}{2}-1} \right]$$

$$=\frac{E\left[ \beta_{t+1} \right]}{\alpha_{0}+\frac{h}{2}-1}$$

$$=\frac{\beta_{0}+\frac{h}{2}\mu_{0}}{\alpha_{0}+\frac{h}{2}-1}$$

$$=\mu_{0}$$

where the simplification between lines 3 and 4 proceeds as before. This concludes the proof.

**Figure 8 – False Positive Rates (FPR) for each model/effect size combination, averaged over MAF.**

**Figure 9 – Overview of the HMC algorithm for neural networks**
